# Adherence to Neuromuscular Electrical Stimulation Interventions for Muscle Impairment in Hip and Knee Osteoarthritis: A Systematic Review

**DOI:** 10.1177/11795441211028746

**Published:** 2021-06-27

**Authors:** Louise C Burgess, Paul Taylor, Thomas W Wainwright, Shayan Bahadori, Ian D Swain

**Affiliations:** 1 Orthopaedic Research Institute, Bournemouth University, Bournemouth, UK; 2 Department of Clinical Science and Engineering, Salisbury District Hospital, Salisbury, Wiltshire, UK; 3 Odstock Medical Limited, Salisbury District Hospital, Salisbury, Wiltshire, UK; 4 Faculty of Health and Social Science, Bournemouth University, Bournemouth, UK; 5 Physiotherapy Department, University Hospitals Dorset NHS Foundation Trust, Bournemouth, UK

**Keywords:** Osteoarthritis, neuromuscular electrical stimulation (NMES), joint replacement surgery, rehabilitation

## Abstract

**Background:**

Neuromuscular electrical stimulation (NMES) provides a promising approach to counteract muscle impairment in hip and knee osteoarthritis, and to expedite recovery from joint replacement surgery. Nonetheless, application into clinical orthopaedic practice remains limited, partly due to concerns regarding patient tolerance.

**Objectives:**

This systematic review aimed to quantify levels of adherence to NMES interventions for muscle impairment in hip and knee osteoarthritis and identify strategies to increase compliance.

**Data Sources:**

Randomised controlled trials (RCTs) were identified in a web-based literature review, completed in December 2020. The databases sourced included the Cochrane Library, CINAHL Complete, Medline Complete and PubMed.

**Eligibility Criteria:**

Studies were included if they were: (i) conducted in cohorts of adults with hip or knee osteoarthritis; (ii) a protocol of electrical muscle stimulation prescribed to treat muscle impairment; and (iii) reported intervention adherence or attrition rate. Data were extracted on adherence rate, reasons for non-adherence and potential strategies to increase adherence. Risk of bias was assessed using the Physiotherapy Evidence Database (PEDro) scale.

**Results:**

The search yielded 120 articles, of which 15 studies were considered eligible and included in the analysis (n = 922). All NMES treatment was applied to the quadriceps, with 1 study targeting the quadriceps and calves. The mean PEDRO score of the included studies was 6.80 out of a possible 10 (range 6-8). Mean adherence did not differ between groups receiving treatment with NMES (85% ± 12%) and control groups receiving voluntary exercise or education (84% ± 9%) (*P* = .97). Reasons for non-adherence or attrition included a dislike of the device, dizziness, pain and discomfort. Strategies to increase adherence included NMES education, a familiarisation period, supervision, setting thresholds based upon patient tolerance, monitoring pain levels during stimulation and using built-in adherence trackers.

**Conclusions:**

This systematic review indicates that adherence to NMES interventions for muscle impairment in hip and knee osteoarthritis in clinical trials does not differ to control groups receiving education or voluntary exercise, and hence should not be a barrier to application in clinical practice.

## Introduction

Osteoarthritis is a chronic debilitating condition that is associated with severe pain, muscle weakness and disability.^1^ In England, it is estimated that 18% of adults aged over 45 years have osteoarthritis of the knee, and 11% have osteoarthritis of the hip.^2^ To counteract musculoskeletal impairment, local muscle strengthening and aerobic exercise are recommended by the National Institute of Health and Clinical Excellence (NICE), in line with international guidelines.^3-6^ Likewise, when progression of the disease leads to consideration for joint replacement surgery, preoperative exercise programmes are proposed as a potential method to expedite recovery time.^7-9^ Nonetheless, many patients avoid voluntary exercise due to fear of exacerbating pain or causing joint damage,^10-14^ and the existing evidence regarding the value of preoperative exercise for patients undergoing joint replacement is conflicting.^7,9^ Furthermore, following surgery, a decrease in voluntary muscle activation can lead to difficult and prolonged rehabilitation.^15^

Neuromuscular electrical stimulation (NMES) is a form of electrical stimulation commonly used at sufficiently high intensities to produce muscle contraction.^16^ With repeated use, NMES can be used as an alternative treatment to counteract muscle impairment in adults with advanced progressive diseases who have difficulty activating their muscles voluntarily.^16^ Therefore, NMES offers unique advantages to preserve or restore skeletal muscle mass and function during and after a period of disuse due to injury, surgery or illness, where voluntary exercise is contraindicated.^17,18^ NMES involves the application of electrical impulses to skeletal muscles, by means of surface electrodes placed over the muscle belly, with the goal of evoking involuntary muscular contractions.^19^ In clinical and performance sport settings, it has been proven to enhance muscle strength, increase range of motion, reduce oedema, prevent atrophy, heal tissue and decrease pain.^20^ However, despite the supporting evidence; NMES remains a clinically underutilised treatment modality in the orthopaedic population.^19^ In addition, in some nations, NMES is not advised in clinical guidelines for hip and knee replacement care, and is therefore only rarely used with orthopaedic patients.^21^ Other reasons for limited adoption include a lack of guidelines on stimulation interventions and parameters, uncertainty regarding the efficacy of stimulation for strengthening muscles and concerns of pain in patients particularly sensitive to electrical stimulation.^19^

New technologies have the potential to revolutionise how we manage health conditions, and recovery from major surgery, both now and in the future. However, successful implementation of new devices can only be achieved once widespread adoption has occurred.^22^ Clinicians can become risk averse and resistant to change if they suspect a new technology is difficult to implement.^23^ The driving force of recent work into NMES has been physiotherapists calling for guidance on effective parameters and application techniques required to achieve optimal results with NMES.^24^ As NMES is a novel therapy modality; understanding patient adherence levels and reasons for non-adherence are important factors that will affect its clinical value and widespread adoption. Moreover, increasing adherence to therapeutic programmes is recognised as an important factor for their long-term effectiveness. The aims of this systematic review are 3-fold: (i) to quantify levels of adherence in NMES interventions for muscle impairment in hip and knee osteoarthritis; (ii) identify reasons for non-adherence and (iii) identify potential strategies to increase adherence.

## Methods

### Protocol and registration

**Figure 1.**
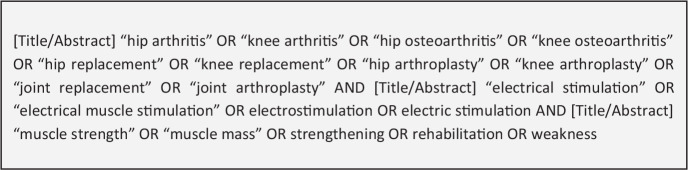
Search strategy.

This is a systematic review, registered a priori on the International Prospective Register of Systematic Reviews (PROSPERO registration number: CRD42020224638) and reported in accordance to the Preferred Reporting Items for Systematic Reviews and Meta-Analyses (PRISMA) statement.^25^ A web-based literature search was completed in December 2020 and the databases sourced included the Cochrane Library, CINAHL Complete, Medline Complete and PubMed, accessed through Bournemouth University’s online library. A search strategy was developed to capture randomised controlled trials (RCTs) of electrical muscle stimulation in adults (over 18 years) diagnosed with hip or knee osteoarthritis (Figure 1). The search reviewed titles and abstracts of the available, peer-reviewed literature published from the earliest record on file until 1st December 2020. Secondary searching was also undertaken; whereby the reference lists of the yielded articles were searched for relevant citations, and to ensure the primary study was selected for inclusion.

### Study selection

Selected studies were screened based on their title and abstract. Once clearly ineligible articles had been removed, full-text screening was conducted by 2 members of the research team (LB and SB). Studies were included if they were: (i) conducted in cohorts of adults with hip or knee osteoarthritis (both the non-surgical and surgical population); (ii) a protocol of electrical muscle stimulation prescribed to treat muscle impairment (NMES or NMES applied functionally, functional electrical stimulation [FES]); (iii) reported adherence (compliance to the study protocol or attrition rate); (iv) available in the English language and (v) peer-reviewed. Studies were excluded if they: (i) prescribed electrical muscle stimulation for reasons other than muscle strengthening (eg, pain relief); (ii) utilised transcutaneous electrical nerve stimulation [TENS]); (iii) prescribed NMES in combination with another strengthening modality other than standard care; (iv) did not report adherence to the electrical stimulation protocol or attrition rate; (v) were a secondary analysis or sub-group analysis of another trial or (vi) were a case-report.

### Data extraction

Data were extracted from the included manuscripts into extraction sheets developed in Microsoft Excel. The following data were extracted: (i) study design; (ii) study population (sample size, type and severity of osteoarthritis); (iii) NMES dose; (iv) adherence to NMES protocol; (v) adherence in the control/comparison group; (vi) study attrition; (vii) reasons for non-adherence (as stated by the authors); (viii) potential strategies to increase adherence (as stated by the authors or considered by the researchers to be a strategy); and (ix) conclusions of the study. If adherence rates were not reported, but the authors reported the number of participants who were non-compliant, a manual calculation was performed by dividing this number by the total number of participants in the trial arm, multiplied by 100. Retention rate was calculated by dividing the attrition rate (dropouts at all time points) by the total number of participants originally enrolled into the trial arm and multiplied by 100. To calculate mean adherence and retention rate across the included studies, each study was given an equal weighting, whereby scores were added together and divided by the number of included studies. In some studies, participants were excluded if they did not meet the target adherence for the study and therefore there is a crossover between the data extracted for study adherence and retention rate. This data is marked with an asterisk in Table 1.

**Table 1.** Summary of included studies.

Study and population	n	Interventions	NMES dose	Comparison intervention (s)	Conclusion (s)	NMES adherence	Comparison adherence	NMES retention	Comparison retention
Klika et al^30^ Knee replacement surgery	66	Postoperative, home-based, unsupervised, app controlled NMES applied to the quadriceps with a knee garment, compared to a control group (standard care).	Duration: postoperative weeks 1-12 Waveform: monophasic Frequency: 50 Hz Pulse duration: 5 ms Duty cycle 25% Current: capable of causing superior patella glide or higher as tolerated. Sessions: 3 per week Time: 20 min	Patients in both arms followed the standard of care physiotherapy regime prescribed by their surgeon, from postoperative day 1 for 12 weeks. Pain management protocols were not standardised and varied by patient and clinical practice.	Use of NMES post-operatively showed significant improvements in quadriceps strength and timed up and go scores, supporting a quicker return to function.	55%	Not reported	55%*	100%
Yoshida et al^31^ Knee replacement surgery	77	Postoperative, supervised sensory level NMES (sNMES) and motor-level NMES (mNMES) of the quadriceps, compared to a control group (standard care).	Duration: postoperative weeks 2-4 Waveform: symmetrical biphasic Frequency: 100 Hz Pulse duration: 1 ms Duty cycle: continuous/10 s on, 10 s off Current: 10-15 mA/maximum tolerated Sessions: 5 per week Time: 45/30 min	All patients received physiotherapy from postoperative day 1 for 4 weeks, including lower extremity exercises, patellofemoral joint mobilisation and ADL exercises. 40-60 min per day, 5-6 days per week.	The mNMES group improved their muscle strength and function significantly more than standard care however reported discomfort. sNMES was more comfortable and led to strength gains.	Not reported	Not reported	sNMES = 88% mNMES = 85%	85%
Melo et al^32^ Knee osteoarthritis	45	Supervised NMES training of the quadriceps compared to laser therapy (LT) and NMES combined with laser therapy (CT) in elderly women.	Duration: 8 weeks Waveform: pulsed symmetric biphasic rectangular Frequency: 80 Hz Pulse duration: 400 μs Duty cycle: not reported Current: max tolerated/40% of MVC Sessions: 2 per week Time: 18-32 min	Laser therapy applied while the probe was held stationary and perpendicular to the skin. Light pressure was applied to 3 anteromedial and 3 anterolateral points over the intercondylar notch. Two times per week, for 8 weeks.	NMES alone or combined with laser therapy increased muscle thickness and cross-sectional area.	Not reported	Not reported	100%	LT = 100% CT = 93%
Levine et al^33^ Knee replacement surgery	70	Unsupervised pre and postoperative NMES training combined with range of motion exercises, compared to conventional, supervised physiotherapy.	Duration: 14 days pre-surgery then days 1-60 postop Waveform: not reported Frequency: not reported Pulse duration: not reported Duty cycle: not reported Current: not reported Sessions: Daily Time: not reported	Patients in the comparison group received a physiotherapy programme including progressive resistive and ROM exercises to be completed whilst hospitalised and after discharge (supervised).	Results did not differ between groups, suggesting that home-based NMES training may provide an option for simplifying and reducing the cost of postoperative physiotherapy.	Not reported	Not reported	80%	71%
Imoto et al^34^ Knee osteoarthritis	100	Supervised quadriceps strengthening exercises and simultaneous NMES treatment compared to a control group receiving education.	Duration: 8 weeks Waveform: pulsed symmetric biphasic rectangular Frequency: 50 Hz Pulse duration: 250 μs Duty cycle: 10 s on, 30 s off Current: maximum tolerated Sessions: not reported Time: 20 min	Education was provided verbally and as a written material. The content included information on knee osteoarthritis, how to adjust ADLs and instructions on applying heat and ice packs if the patient experienced swelling or soreness.	NMES in this rehabilitation programme was effective for improving pain, function and ADLs, in comparison with a group that received education only.	90%	Not reported	88%*	76%
Bruce-Band et al^35^ Knee osteoarthritis	41	Unsupervised NMES training of the quadriceps compared to resistance training (RT) and a control group (CG).	Duration: 6 weeks Waveform: symmetrical biphasic square Frequency: 50 Hz Pulse duration: between 100-400 μs Duty cycle: 10 s on, 50 s off Current: maximum tolerated Sessions: 5 per week Time: 20 min	RT – 3 session per week, for 6 weeks (approx. 30 min). Patients were supplied with a logbook of lower limb exercises such as leg raises and wall squats (3 sets, 10 reps). CG – Standard care included education, weight loss, pain relief and physiotherapy.	Home-based NMES was an acceptable alternative to exercise therapy, producing similar improvements in functional capacity.	91%	RT = 83% CG = not reported	71%	RT = 71% CG = 46%
Elboim-Gabyzon et al^36^ Knee osteoarthritis	63	Supervised NMES training of the quadriceps plus group exercise compared to group exercise alone.	Duration: 6 weeks Waveform: biphasic Frequency: 75 Hz Pulse duration: 200 µs Duty cycle: 10 s on, 50 s off Current: maximum tolerated Sessions: 2 per week Time: 10 contractions	Group exercise and education sessions included ROM and lower extremity muscle strengthening exercises, functional activities and balance training. 45 min sessions, conducted biweekly for 6 weeks (12 sessions).	NMES improved voluntary activation in patients with knee osteoarthritis but did not enhance its effect on muscle strength or function.	90%	79%	83%*	76%*
Stevens-Lapsley et al^37^ Knee replacement surgery	66	Standard, supervised, postoperative rehabilitation combined with NMES of the quadriceps, initiated 48 h after surgery, compared to standard rehabilitation.	Duration: 6 weeks Waveform: symmetrical biphasic Frequency: 50 Hz Pulse duration: 250 µs Duty cycle: 15 s on, 45 s off Current: maximum tolerated Sessions: 2 per day, 6-7 days per week Time: 15 contractions	Standard rehabilitation included passive knee ROM, patellofemoral mobilisation, cycling, flexibility exercises, ice and heat if needed, gait training, functional and resistance training.	The early addition of NMES effectively attenuated loss of quadriceps muscle strength and improved functional performance.	77%	Not reported	86%	81%
Walls et al^38^ Knee replacement surgery	17	Preoperative, unsupervised, home-based NMES training of the quadriceps with a knee garment, compared to standard preoperative care.	Duration: 8 weeks Waveform: symmetrical biphasic Frequency: 50 Hz Pulse duration: between 100-400 μs Duty cycle: 5 s on, 10 s off Current: maximum tolerated Sessions: Every other day for 2 weeks, then 5 days per week. Time: 20 min	Individualised instructions on knee ROM and quadriceps strengthening exercises from a physiotherapy, for example, static quads and leg raises. Sets of 10-20 reps for each exercise, 2 × per day.	Preoperative NMES may improve quadriceps muscle strength recovery and expedite a return to normal function in patients undergoing knee replacement.	99%	Not reported	82%	83%
Palmieri-Smith et al^39^ Knee osteoarthritis	30	Supervised NMES training of the quadriceps delivered to women with radiographic mild to moderate osteoarthritis compared to a control group (standard care [no treatment]).	Duration: 4 weeks Waveform: alternating current Frequency: 50 Hz Pulse duration: not reported Duty cycle: 10s on, 50s off Current: maximum tolerated or at least 35% of MVC Sessions: 3 per week Time: 10 contractions	No intervention, as this is considered standard of care for those currently not seeking treatment for osteoarthritis.	Four weeks of NMES training was insufficient to induce gains in quadriceps muscle strength or activation.	88%	Not reported	69%	57%
Petterson et al^40^ Knee replacement patients	200	Supervised postoperative NMES training of the quadriceps and voluntary strength training, starting 2-4 weeks post-surgery, compared to an exercise group (EG) and control group who agreed to be tested 12 months post-op.	Duration: 6 weeks Waveform: sinusoidal, alternating Frequency: 50 Hz Pulse duration: not reported Duty cycle: 10 s on, 80 s off Current: max tolerated or 30% of MVC Sessions: 2-3 per week Time: 10 contractions	Both groups received outpatient physiotherapy 2-3 times per week, for 6 weeks. Interventions targeted knee extension and flexion ROM, patellar mobility, quadriceps strength, pain control and gait. 2 × 10 reps/sets progressed to 3 × 10. Weights were added to add intensity.	Progressive quadriceps strengthening with or without NMES enhances clinical improvement after knee replacement surgery, achieving similar short and long-term functional recovery.	84%	EG = 97% CG = N/A	68%	EG = 81% CG = N/A
Gremeaux et al^41^ Hip replacement surgery	29	Postoperative, supervised NMES training of the quadriceps and calves combined with conventional physiotherapy in elderly patients, compared to standard care.	Duration: 5 weeks Waveform: biphasic Frequency: 10 Hz Pulse duration: 200 μs Duty cycle: 20s on, 20s off Current: maximum tolerated Sessions: 5 per week Time: 60 min	Both groups received conventional physiotherapy including exercise to increase joint ROM, muscle strength, functional status and cardiovascular conditioning. 2 h per session, 5 × per week (25 sessions).	Low-frequency stimulation improved knee extensor strength, which is one of the factors leading to greater functional independence after hip replacement.	Not reported	Not reported	100%	81%
Durmus et al^42^ Knee osteoarthritis	50	Supervised NMES training of the quadriceps, compared to biofeedback-assisted isometric exercises, in an outpatient department.	Duration: 4 weeks Waveform: asymmetric biphasic Frequency: 50 Hz Pulse duration: 200 μs Duty cycle: 10 s on, 10 s off Current: to establish apparent muscle contraction Sessions: 5 per week Time: 20 min	Biofeedback-assisted exercise whereby patients were asked to perform isometric quadricep contractions for 10 s with 50 s relaxation. The patient was asked to increase visual and auditory signals that they perceived at every contraction.	NMES was as effective as exercise in treating knee osteoarthritis and may be considered for those who have difficulty in or contraindications to voluntary exercise.	Not reported	Not reported	100%	100%
Talbot et al^43^ Knee osteoarthritis	38	Home-based NMES training of the quadriceps combined with education, compared to education alone.	Duration: 12 weeks Waveform: symmetrical biphasic rectangular Frequency: 50 Hz Pulse duration: 300 μs Duty cycle: 10 s on, 50 s off Current: max tolerated or progressed from 10%-40% MVC Sessions: 3 per week Time: 15 min of 15 stimulations	Arthritis self-help course, once a week for 12 weeks. The programme taught disease aetiology, self-management techniques and goal setting. Leaders were 2 nurses.	Home-based NMES in older adults with knee osteoarthritis demonstrated promising effects to knee extensor strength, chair rise ability and walk speed, without exacerbating painful symptoms.	81%	78%	90%	89%
Oldham et al^44^ Knee osteoarthritis	30	A comparison of unsupervised patterned NMES, random pattern NMES, uniform stimulation and sham NMES in elderly patients on the waiting list for TKR.	Duration: 6 weeks Waveform: asymmetrical biphasic Frequency: patterned stimulation/random interpulse intervals/uniform frequency of 8.4 Hz Pulse duration: 300 μs Duty cycle: 30 s on, 15 s off Current: minimum required to produce both visible and palpable muscle contraction Sessions: daily Time: 3 h	The sham stimulation group received stimulation comprising a single 300 μs impulse every 3 min.	No stimulation pattern emerged as being significantly better than another, although statistically significant differences between individual stimulation patterns were observed at a number of assessment weeks.	90%	Not reported	Two patients dropped out, but it is not clear which group they were in.	

Abbreviations: ADL, activities of daily living; CG, control group; CT, combined therapy; EG, exercise group; LT, laser therapy; mNMES, motor-level NMES; MVC, maximal voluntary contraction; NMES, neuromuscular electrical stimulation; ROM, range of motion; RT, resistance training; sNMES, sensory level NMES.

* Non-compliance used as a criterion for exclusion/drop-out.

### Data synthesis

The characteristics of the included studies were presented using a descriptive analysis. Mean adherence and retention rates were compared between the participants prescribed an intervention of NMES and the control/comparison group. Furthermore, mean adherence and retention rates were compared between patients who received supervised and unsupervised NMES, and between surgical and non-surgical patients. The normality of this data was evaluated using a Shapiro-Wilk test. All data were normally distributed, and hence, unpaired *T*-tests were used to evaluate the relationship between groups. A Pearson’s Correlation was used to investigate any relationship between duration of NMES intervention, and adherence and retention. All data were analysed using IBM SPSS Statistics version 26 (SPSS Inc., Chicago, USA), with the significance level set at *P* < .05. Correlation coefficients were interpreted using definitions from Chan.^26^ Qualitative data on reasons for non-adherence and strategies to increase adherence were summarised and presented descriptively.

### Quality assessment

The PEDro (Physiotherapy Evidence Database) scale was used to critically appraise the studies included within our search.^27^ The methodological quality of the studies was determined independently by 2 members of the research team (LB and SB) and discrepancies were resolved through discussion with the wider research team. The 11 item scale is a valid measure used to assess clinical trials,^28,29^ with each study scored out of 10; with a score of 6 as the threshold for a high-quality study (item 1 on the scale indicates external validity). The PEDro scale scores 10 items; random allocation, concealed allocation, similarity at baseline, subject blinding, therapist blinding, assessor binding, greater than 85% follow up for at least 1 key outcome, intention-to-treat analysis, between group statistical comparison for at least 1 key outcome and point and variability measures for at least 1 key outcome.^28^

## Results

**Figure 2.**
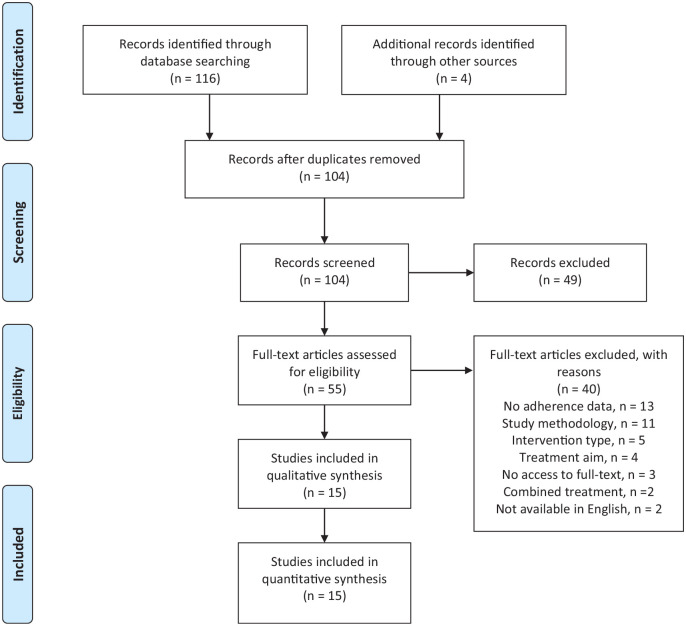
Study identification flowchart.^23^

The search yielded 116 articles, and an additional 4 were sourced through secondary searching (Figure 2). Once duplicates (n = 16) were removed, the titles and abstracts of the remaining 104 results were screened for eligibility. Following the removal of clearly ineligible studies (n = 49), the remaining 55 studies underwent full-text screening. A further 40 studies were removed for the following reasons: did not report adherence or attrition rate (n = 13); excluded study type, or was a secondary analysis of an included study (n = 11); excluded treatment type (n = 5); excluded treatment aim (n = 4); no access to full-text (n = 3); combined treatment approach (n = 2) and not available in the English language (n = 2). Fifteen studies were considered eligible and included in the final analysis (Table 1).^30-44^

### Characteristics of included studies

Fourteen of the yielded studies were randomised controlled trials^30-37,39-44^ and 1 was a pilot randomised controlled trial,^38^ published between 1995 and 2020. The mean PEDro score of the included studies was 6.80 out of a possible 10 (range 6-8), corresponding to a high level of internal validity (Table 2).^45^ Consistently low scoring items were criterion 5 and 6, blinding of subjects and therapist. The study that compared NMES to sham stimulation was the only study that was awarded a point for item 5.^44^ Other low scoring items were criterion 7 (assessor blinding) and 8 (measures of at least 1 key outcome obtained from more than 85% of the subjects initially allocated to the group).

**Table 2.** Grade of evidence PEDro score. The circle represents the study being awarded a point for each criterion of the PEDRro scale.

Study	n	1. Eligibility criteria	2. Random allocation	3. Concealed allocation	4. Similar groups	5. Subject blinding	6. Therapist blinding	7. Assessor blinding	8. 85% outcomes	9. Intention to treat	10. Outcome comparison	11. Variability measures	PEDro score out of 10
Klika et al^30^	66	•	•	•	•			•		•	•	•	7
Yoshida et al^31^	77	•	•	•	•			•	•	•	•	•	8
de Oliveira Melo et al^32^	45	•	•	•	•			•	•	•	•	•	8
Levine et al^33^	70	•	•	•	•				•	•	•	•	7
Imoto et al^34^	100	•	•	•	•			•		•	•	•	7
Bruce-Brand et al^35^	41	•	•	•	•			•		•	•	•	7
Elboim-Gabyzon et al^36^	63	•	•	•	•					•	•	•	6
Stevens-Lapsley et al^37^	66	•	•	•	•				•	•	•	•	7
Walls et al^38^	17	•	•	•	•			•		•	•	•	7
Palmieri-Smith et al^39^	30	•	•	•	•		•		•	•	•	•	8
Petterson et al^40^	200	•	•		•			•		•	•	•	6
Gremeaux et al^41^	29	•	•		•				•	•	•	•	6
Durmus et al^42^	50	•	•		•				•	•	•	•	6
Talbot et al^43^	38	•	•		•				•	•	•	•	6
Oldham et al^44^	30	•	•			•		•	•	•	•		6

### Sample characteristics

A total of 922 participants were included in the studies, 475 of which were enrolled into an intervention of NMES that aimed to increase muscle strength or reduce atrophy. Six of the studies were conducted with patients undergoing knee replacement,^30,31,33,37,38,40^ 8 were with non-surgical knee osteoarthritis patients,^32,34-36,39,42-44^ and 1 study included patients listed for hip replacement surgery.^41^ Treatment with the surgical arthritic population was typically postoperative, however 1 study investigated preoperative NMES, initiated 8 weeks prior to surgery,^38^ and 1 study was initiated 14 days pre-surgery and continued for 60 days following surgery.^33^ In the non-surgical articles, 2 studies included patients with mild-to-moderate symptoms,^32,39^ 1 study included patients with moderate-to-severe symptoms,^35^ 1 study included patients with end-stage osteoarthritis^44^ and 4 studies included a mixed sample.^34,36,42,43^

### Intervention characteristics

Studies were a combination of home-based, unsupervised NMES and supervised NMES, delivered in a hospital or a physiotherapy clinic. The studies compared a programme of NMES to a control group receiving no treatment,^39^ conventional physiotherapy care,^30,31,33,35,37,38,40,41^ voluntary exercise,^35,36,40,42^ laser therapy,^32^ education only^34,43^ or sham stimulation.^44^ Two studies compared NMES to a control group and an exercise group.^35,40^ Voluntary exercise interventions included partially supervised, home-based resistance training,^35^ supervised group exercise including lower-extremity strengthening, range of motion exercise, functional activities and balance training,^36^ volitional strength training targeting the quadriceps at an outpatient physiotherapy department^40^ and biofeedback assisted isometric contractions.^42^ Standard postoperative care varied between studies, but generally included lower extremity strengthening exercise, range of motion exercises, patellofemoral mobilisation (following knee replacement only), gait training and exercises related to activities of daily living. Education groups received information on adjusting their daily living according to their symptoms,^34^ and an arthritis self-help course, including details on disease aetiology, self-management techniques and goal setting.^43^

Studies ranged from 2 to 12 weeks in duration, with a median length of 6 weeks. All studies targeted the quadriceps femoris muscle group, with 1 study stimulating the quadriceps and calves.^41^ Two studies investigated more than 1 type of NMES. In the study by Yoshida et al^31^ sensory level NMES and motor-level NMES were compared to a control group. Oldham et al^44^ compared patterned NMES, random patterned NMES and uniformed stimulation to sham NMES.

Use of NMES was reported to improve quadriceps strength,^30,31,33,38,40-44^ voluntary quadriceps activation,^36^ muscle thickness and cross-sectional area,^32^ muscle atrophy,^37^ pain^34^ and functional outcome measures^30,31,33-35,37,38,42-44^ however did not enhance muscle activation,^39^ strength^36,39^ or function^36^ in 2 studies. The main conclusions from the studies are described in Table 1.

### Definitions of adherence

Data on adherence were extracted from 10 studies, and data on study attrition from 14 (Table 1). For unsupervised NMES, adherence was commonly defined as the total stimulation time recorded by the device tracker or in the participant logbook, divided by the total dose prescribed and multiplied by 100. For supervised stimulation, adherence was defined as the number of sessions attended divided by the total sessions, multiplied by 100. In 3 studies, adherence was compared between the device tracker and the participant logbook. Complete concordance was found in 2 studies^35,38^ and in 1 study, the device tracker suggested a higher use than that recorded in the logbook.^43^

### Adherence

Mean adherence in the NMES group was 85% ± 12% (range: 55%-99%), and 84% ± 9% (range: 78%-97%) in the comparison groups receiving exercise or education. Retention rate in the NMES group was 83% ± 13% (range: 55%-100%) and 81% ± 15% in the patients receiving standard care, laser-therapy, sham stimulation, education or voluntary exercise (range: 46%-100%). There were no differences between the NMES and comparison/control groups in terms of adherence (*P* = .97) or retention rate (*P* = .64).

Mean adherence for those receiving supervised NMES was 86% ± 6% (range: 84%-90%), and 83% ± 17% (range 55%-91%) for those receiving unsupervised NMES (*P* = .76). Mean retention rate for those receiving supervised NMES was 87% ± 12% (range 68%-100%), and 76% ± 13% (range: 55%-90%) for those receiving unsupervised NMES (*P* = .16).

Mean adherence for surgical patients was 79% ± 18% (range: 55%-99%) whereas non-surgical patients had a mean adherence rate of 88% ± 4% (range 81%-90%) (*P* = .37). Mean retention rate for surgical patients was 81% ± 14% (range: 55%-100%), and 86% ± 12% (range 69%-100%) for non-surgical patients (*P* = .44).

Pearson’s correlation coefficient demonstrated a moderate, negative relationship between duration of treatment and adherence rate (*r* = −.57, *P* = .08) and a weak, negative relationship between duration of treatment and retention rate (*r* = −.26) that also did not reach significance (*P* = .38). This may be due to the small sample included within the correlation analysis.^46^

### Strategies to increase adherence

Preoperative education and a familiarisation period were highlighted as potential contributors to protocol adherence.^30,37^ In addition, it was speculated that supervision, or an additional home-training session to ensure safety and encourage tolerance helped to increase adherence.^34,37^ In the study by Bruce-Brand et al,^35^ the relative simplicity of the NMES protocol, combined with the novelty of the modality and the built-in tracker were discussed as potential reasons for high adherence. High adherence in the study by Walls et al^38^ was attributed to the simplicity of garment based NMES compared to application through electrodes. However, in the study with the lowest level of adherence, NMES was also applied through a knee garment.^30^

To monitor and increase adherence the studies included: comprehensive NMES training,^35^ written instructions to use devices in the home environment,^35^ a clear training programme schedule,^38^ an intensity threshold set to suit patient tolerance,^30^ built-in adherence monitors^30,32,37,38,43,44^ and participant logbooks.^33,35,37,38,43,44^ In some studies, participants were aware of the built-in adherence monitor,^30,37,43,44^ and in some cases, participants did not know that their adherence was being tracked.^38^ Logbooks collected data on the dates and duration of the NMES sessions, amplitude settings, rate of perceived exertion and level of pain. In 1 study with surgical patients, an initial familiarisation period was used preoperatively to facilitate postoperative utilisation, and patients were required to demonstrate safe and proper use in-hospital prior to discharge.^37^ In home-based interventions, some participants were visited at home to monitor an independent treatment session, to assess procedural reliability.^37,40^ This was either done routinely, or in cases where concerns arose about participant implementation or tolerance to NMES. In the study by Stevens-Lapsley et al,^37^ marking the electrode locations on the thigh was thought to ensure proper electrode placement, which may help increase treatment adherence and fidelity. Furthermore, an emphasis was placed on the importance of using the stimulator at an intensity that was tolerable but slightly uncomfortable.^37^ To increase treatment fidelity, in 1 study, if the self-selected intensity did not result in visible contractions, the participant was excluded from the trial.^31^ In the study by Gremeaux et al,^41^ the degree of pain related to the stimulation was monitored every 5 sessions using a 6 level verbal scale. A score of 3 or higher resulted in exclusion from the protocol.

### Reasons for non-adherence

Participants who were non-compliant reported that they did not like the device or did not want to be inconvenienced whilst recovering from surgery.^30^ Other reasons for non-adherence and attrition related to the device included discomfort, dizziness and pain.^31,36,40^ In the study by Stevens-Lapsley et al,^37^ the authors discussed how therapists may be reluctant to push patients to tolerate uncomfortable doses of stimulation which may limit the potential benefits of the treatment. As such, the authors suggest that education regarding tolerating maximum doses of stimulation is important.^37^

## Discussion

Rates of hip and knee osteoarthritis, and joint replacement surgeries, are predicted to increase in line with the ageing population and the global obesity epidemic.^47^ As the National Health Service (NHS), along with health services across the globe, face rising capacity and funding challenges, the UK government has looked towards the possible benefits of new technologies to improve productivity and patient outcomes.^48^ However, successful implementation of new technologies can only be achieved once widespread adoption has occurred.^22^ To date, application of NMES into clinical orthopaedic practice has been slow, despite the increasing scientific evidence to support its effectiveness for treating muscle impairment.^24^ Recent research has been driven by physiotherapists calling for further guidance on effective parameters and application techniques required to achieve optimal results with NMES.^24^ This review provides a synthesis of evidence for adherence to NMES interventions for muscle impairment in the hip and knee osteoarthritis population, and to our knowledge, is the first of its kind. We have identified strategies that may increase adherence when prescribing NMES and highlighted potential reasons for non-adherence. Perhaps most interestingly, we found that adherence to the prescribed treatment did not differ between groups receiving treatment with NMES and control groups receiving education or voluntary exercise. Furthermore, there were no differences in retention rates between the NMES group and patients receiving standard care, laser-therapy, sham stimulation, education or voluntary exercise. These findings are promising, given the concern that NMES may not be an acceptable treatment for patients particularly sensitive to electrical stimulation.^19^

Our findings may encourage clinicians to consider providing comprehensive NMES training, written instructions on how to use the device, a training schedule and an initial familiarisation period when prescribing NMES treatments. We also found that using patient logbooks or built-in trackers will likely encourage adherence. Adherence and retention rates amongst supervised NMES interventions were higher than unsupervised interventions, although these relationships were not significant. Likewise, non-surgical patients had higher adherence and retention rates than non-surgical patients, but these relationships were also non-significant. Potential reasons for non-adherence in NMES treatments included a dislike of the device, dizziness, pain and discomfort. Strategies to counteract these reasons could involve monitoring pain levels during stimulation and setting intensity thresholds based upon patient tolerance. However, to be effective in treating muscle impairment, stimulation intensity needs to be high enough to evoke an involuntary muscle contraction,^49^ and although device trackers allow clinicians to observe total usage, it is not always possible to monitor stimulation intensity. Nonetheless, promising evidence was found in the study by Palmieri-Smith et al,^39^ where stimulation intensity was evaluated during supervised treatment. Participants were able to tolerate stimulation at an intensity sufficient to achieve the target contraction strength (35% MVC or greater) in 93% of the treatment sessions.^39^

Whilst this research is novel in the area of NMES, several reviews have evaluated adherence to voluntary exercise in patients with hip and knee osteoarthritis.^50-54^ One review found that just 33% of patients were fully adherent to an exercise programme prescribed following completion of the supervised element of the programme, and 37% were partially adherent.^53^ Likewise, in a study by Pisters et al^55^ adherence within the 3 months treatment period was reported at 57.8%, but reduced to 44.1% and 30.1% at 15 and 60 months follow up, respectively. Traditional exercise for patients chronic musculoskeletal disease can be painful, and thus adherence to voluntary exercise often reduces over time.^56^ Likewise, immediately following joint replacement surgery, a decrease in voluntary muscle activation can lead to difficult and prolonged rehabilitation. Nonetheless, therapy is necessary due to significant weakness noted in the musculature in patients with lower-limb osteoarthritis and following joint replacement surgery.^15,57,58^ The findings from this review suggest that adherence to NMES interventions may, in some cases, be higher than adherence to voluntary exercise interventions, and therefore provide promising results for clinicians considering treatment with NMES.

The integration of technology-based exercise programmes may have a positive effect on adherence as they can overcome perceived barriers to exercise,^59^ however, must be prescribed to the right patients, in the optimal therapeutic window, with evidence-based dosing. Some patients with osteoarthritis will be contraindicated to voluntary exercise due to significant joint damage, recent joint replacement surgery or comorbidities, such as cardiac disease or hypertension.^60^ Other patients may experience psychological or behavioural restrictions to voluntary exercise, such as concerns surrounding their capability to exercise, a fear of pain aggravation, along with time, transport and access restraints.^10-12^ Where voluntary exercise is inhibited by pain during joint loading, NMES can be used as an alternative approach to prevent atrophy or strengthen weakened musculature. In addition, NMES offers an innovative approach to mitigate voluntary activation deficits and prevent atrophy early after surgery where a patient may be unable to generate muscle contractions of sufficient intensity to promote strength gains.^37^ However, successful clinical outcomes depend upon patients’ adherence to a prescribed treatment regimen,^61^ and if clinicians are unsure that NMES is an acceptable treatment for patients with osteoarthritis, they may avoid prescribing it. This review found that adherence to NMES interventions for muscle impairment in hip or knee osteoarthritis does not differ to conventional physiotherapy treatments and therefore provides promising results for future clinical use. We recommend that clinicians consider the strategies identified in this review to increase adherence to NMES interventions. Future research endeavours may consider investigating optimal NMES prescription amongst orthopaedic patients, to further increase clinical adoption.

## Limitations

While this review provides a summary of adherence levels to NMES interventions in research studies, estimates derived from clinical trials differ from the actual levels of adherence in the context of clinical practice, where adherence may be much lower. In addition, the analysed studies were heterogeneous, predominantly concerning patient population, sample size, comparison interventions and methods of calculating adherence. Finally, it should be considered that reasons for non-adherence and study attrition may not always be related to the success or failure of the intervention itself. For example, some patients dropped out of the research trials due to medical necessity or family commitments.

## Conclusions

Despite the supporting evidence, NMES remains a clinically underutilised treatment modality in the orthopaedic population, partly due to concerns regarding patient tolerance. This systematic review indicates that adherence to NMES interventions used to increase muscle strength or reduce atrophy in hip and knee osteoarthritis does not differ to control groups receiving education or voluntary exercise in clinical trials, and hence should not be a barrier to application in clinical practice. Reasons for non-adherence or attrition may include a dislike of the device, dizziness, pain and discomfort. Strategies to increase adherence to NMES interventions may include NMES education, a familiarisation period, setting intensity thresholds based upon patient tolerance, built-in adherence trackers, monitoring pain levels and supervision of patients during stimulation.
